# Spontaneous Cervical Artery Dissection: The Borgess Classification

**DOI:** 10.3389/fneur.2013.00133

**Published:** 2013-09-17

**Authors:** Firas Al-Ali, Brandon C. Perry

**Affiliations:** ^1^Neurointerventional Surgery and Diagnostic Services, Borgess Medical Center, Kalamazoo, MI, USA; ^2^College of Human Medicine, Michigan State University, East Lansing, MI, USA

**Keywords:** stroke, cerebral arteries, vertebral artery, dissection, pathophysiology

## Abstract

**Background and Purpose:** The pathogenesis of spontaneous cervical artery dissections (sCAD) and its best medical treatment are debated. This may be due to a lack of clear classification of sCAD. We propose the new Borgess classification of sCAD, based on the presence or absence of intimal tear as depicted on imaging studies and effect on blood flow.

**Materials and Methods:** This is a single-center investigator-initiated registry on consecutive patients treated for sCAD. In the Borgess classification, type I dissections have intact intima and type II dissections have an intimal tear.

**Results:** Forty-four patients and 52 dissected arteries were found. Forty-nine of 52 dissections (93%) were treated with dual anti-platelet therapy. Twenty-one of 52 dissections were type I; 31 were type II. Type I dissections were more likely to present with ischemic symptoms [stroke, transient ischemic attack (TIA)] (*p* = 0.001). More type I dissections occurred in the vertebral artery, while more type II dissections occurred in the internal carotid artery (*p* < 0.001). Follow-up averaged 18.1 months (range: 3–108 months) with no recurrent ischemic events (stroke, TIA), deaths, or hemorrhage. Forty-six vessels had 6 month follow-up on medical treatment; 19/46 (41%) healed. Type I dissections were more likely to heal than type II (*p* < 0.001).

**Conclusion:** The two dissection types in the Borgess classification appear to relate to clinical presentation and rate of healing, making the classification useful in clinical management. Dual anti-platelet therapy for sCAD seems to have a very low risk of subsequent stroke; however, a large prospective study is needed to investigate the best treatment.

## Introduction

Despite the fact that spontaneous cervical artery dissection (sCAD) is relatively common and easily diagnosed using modern imaging techniques, its pathophysiology is still surprisingly poorly understood and its treatment still debated (1–6). Two common explanations describe its pathogenesis: inside-out and outside-in theories. The traditional inside-out theory suggests that a tear in the intima is the primary insult allowing for blood to enter the vascular wall. The newer outside-in theory proposes that an intramural hematoma forms first with or without subsequent intimal tear. The proposed treatment strategies for sCAD are even more complex. The ongoing Cervical Artery Dissection In Stroke Study (CADISS) has six different treatment groups (aspirin, dipyridamole, clopidogrel, dual anti-platelet, unfractionated heparin bridged to warfarin, and low-molecular-weight heparin bridged to warfarin) (6). Furthermore, a few authors have begun to advocate endovascular approaches as a first-line of treatment (7–10). We believe this confusion concerning sCAD pathophysiology and treatment is due, at least in part, to the poor definition and classification of the disease process.

The Borgess classification presented here is based on review of imaging findings of the Borgess Medical Center Spontaneous Cervical Arterial Dissection Registry (BMC-sCAD) at presentation, throughout the patients’ clinical course, and evolution on follow-up. We hope that this new classification will allow for easier and more precise descriptions of sCAD that may impact treatment recommendations and improve prediction of long-term prognosis.

## Borgess Classification of sCAD

The defining feature of the Borgess classification of sCAD is the presence or absence of an intact tunica intima in the dissected vessel and its impact on blood flow, as assessed using available imaging techniques. Intimal tear was considered present if the imaging study demonstrated contrast pooling outside the vascular lumen (side-wall aneurysm), false lumen with intimal flap, or if there was a fusiform dilatation of the vessel. The impact on the cerebral flow was assessed by the persistence (yes or no) of blood flow reaching the intra-cranial circulation.

**Figure 1:**
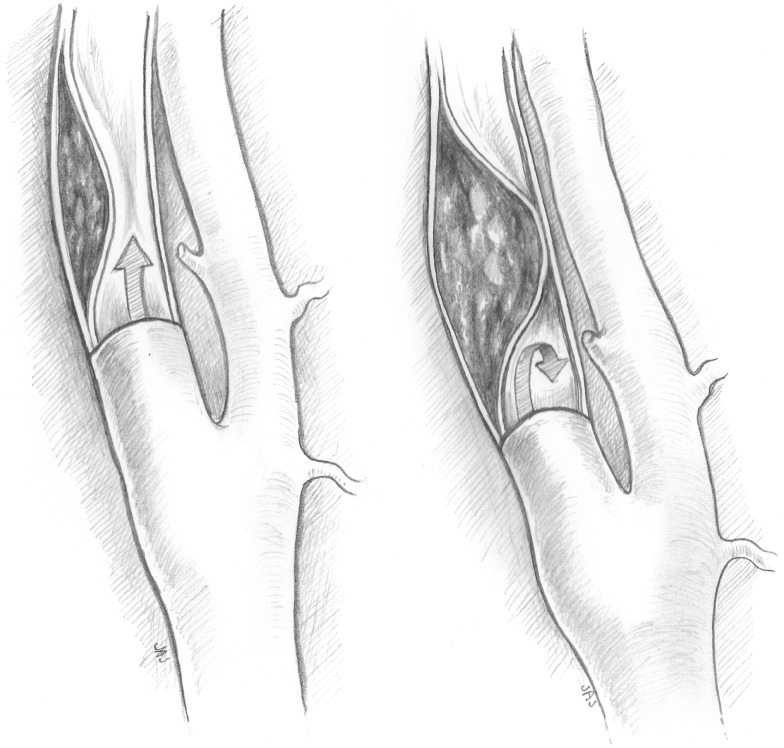
**Type IA and type IB dissections**. Type I dissections show an intact intima. Type IA (left) has persistent antegrade flow. Type IB (right) is completely occluded.

**Figure 2:**
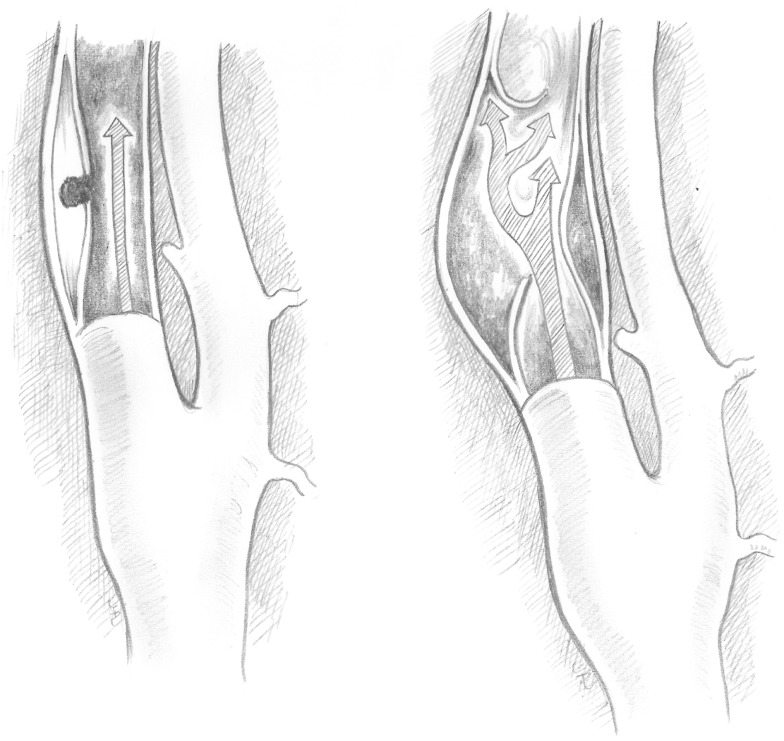
**Type IIA and type IIB dissections**. Type II dissections show intimal disruption. Type IIA (left) has a small disruption of the intima with a small side-wall aneurysm. Type IIB (right) shows a clear intimal flap and aneurysmal dilation.

Type I dissections have an intact intima while type II dissections have a disruption of the intima; both type I and type II are divided into two subtypes (Figures 1 and 2). Type IA dissections show varying degrees of luminal stenosis due to an intramural hematoma, but antegrade flow persists. Type IB dissections have total occlusion of the lumen due to the intramural hematoma with absence of antegrade flow. Type IIA dissections have a small focal disruption of the intima with development of a side-walled aneurysm (the tunica adventitia is intact so the term pseudoaneurysm is inaccurate) of varying size and flow stagnation within the aneurysm. Type IIB dissections have a large disruption of the intima, with either false lumen (intimal flap can be seen and the false lumen may or may not reconnect with the original lumen) or aneurysmal dilation of the affected vessel.

## Materials and Methods

### Study design and subject eligibility

Borgess Medical Center is a high-volume acute ischemic stroke center, treating approximately 300 stroke and 300 transient ischemic attack (TIA) cases per year. The BMC-sCAD is a non-randomized, single-center, single-operator, investigator-initiated registry of all consecutive patients presenting to our service with sCAD between January 2002 and December 2010. All patients gave written informed consent for the procedures. The protocol for this registry was approved by the Institutional Review Board, including approval to waive informed consent for the data collection and analysis. Data were entered prospectively and retrospectively in a secure database (MD Analyze; Medtech Global, South Melbourne, VIC, Australia; www.medtechglobal.com) and then analyzed retrospectively.

Patients suffering from iatrogenic dissections and patients with acute ischemic stroke (within 6 h of ictus) who were treated with the acute ischemic stroke protocol (intra-arterial or intra-venous) were excluded. Diagnosis of sCAD was based on clinical signs of local symptoms (as defined below) or cerebral ischemia and confirmed by at least one imaging study including magnetic resonance angiography (MRA), computed tomography angiography (CTA), or diagnostic cerebral angiography (DCA). Both internal carotid artery (ICA) dissections and vertebral artery (VA) dissections were included. Dissection was classified as spontaneous when occurring unexpectedly or secondary to a usual event such as abrupt neck movement, minor neck or head trauma, chiropractic manipulation, or coughing.

Per protocol, patients were started on dual anti-platelet therapy consisting of clopidogrel (75 mg daily) after a loading dose of 300 mg and aspirin (81 mg daily) immediately after imaging confirmation of dissection. The presence of a cortical infarct did not preclude the initiation of dual anti-platelet therapy. Endovascular treatment was not performed as first-line treatment, except in a few early cases due to confusion about the best treatment modality to decrease the patient’s risk of subsequent stroke. All patients were instructed to contact the neurointerventional service if they experienced any symptoms suggestive of new or recurrent local symptoms or cerebral ischemia. All patients were required to have imaging and clinical follow-up at a minimum of 3 month intervals until resolution or interval progression of the dissection was documented by MRA, CTA, or DCA. Endovascular treatment was offered if follow-up visits documented persistence of local symptoms or the enlargement/persistence of a previously documented large aneurysm.

### Data collection

Demographics (age, gender, and race), the clinical presentation upon admission, and the location of the dissections were collected prospectively. Clinical presentation was divided into three categories: stroke, TIA, and local signs/symptoms. Stroke was identified by clinical symptoms and confirmed by either MRI or CT scans, defined as diffusion-weighted image abnormality on MRI or hypodensity on CT. TIA was defined as a temporary episode of neurologic dysfunction without evidence of infarction on imaging. Local signs and symptoms included neck pain, headache, Horner’s syndrome, and tinnitus. If patients had ischemic and local symptoms, they were classified under ischemic symptoms.

Retrospectively collected data included the type of dissection (type IA, IB, IIA, or IIB) this was obtained from reviewing the imaging studies. Intimal tear was considered present (Borgess Type II) if signs of contrast material were present outside the lumen on DCA or CTA (or flow related signal on the MRA). Sixteen vessels had only MRA available 31% (16/52). Other data collected retrospectively included the presence or absence of vessel kinking, and fibromuscular dysplasia (FMD). Reconstitution of the vessel lumen to its expected size, with resolution of the side-wall aneurysm (if present), was considered complete healing. Side-wall aneurysm was considered small if its longest diameter was equal to or smaller than the diameter of the parent vessel.

### Statistical analysis

Equality of proportions was tested using a 2-Proportion *z*-test. If a low number of successes existed in the data, Fisher’s Exact test was used instead. The predictive value of the variables for type I or type II was examined using binary logistic regression. All hypothesis tests were conducted at the 0.05 significance level using the Minitab 16 statistical software.

## Results

From January 2002 through December 2010, 44 patients and 52 dissected arteries were found. Diagnosis was established in all patients by at least one imaging study. Data stratified by dissected vessel is summarized in Table 1.

**Table 1:** **Characteristics of carotid and vertebral artery dissections**.

	Carotid arterydissection *n* = 38 (%)	Vertebral arterydissection *n* = 14 (%)
**DEMOGRAPHICS**		
Women	19 (50)	8 (57)
Men	19 (50)	6 (43)
**AGE**		
Mean	52.3	44.8
**VESSEL DISSECTED**		
Left	25 (66)	8 (57)
Right	13 (34)	6 (43)
**CLINICAL PRESENTATION (PER VESSEL)**		
Ischemia	13 (34)	8 (57)
Local	14 (37)	6 (43)
Asymptomatic	8 (21)	0 (0)
Non-specific	3 (8)	0 (0)
**DIAGNOSTIC METHOD**		
MRA	30 (52)	10 (43)
CTA	6 (10)	3 (14)
DSA	22 (38)	10 (43)
**INITIAL IMAGING FEATURES PRESENT**		
Fibromuscular dysplasia	15 (39)	3 (21)
Vessel kinking	15 (39)	7 (50)
Type I dissection	10 (26)	11 (79)
Type II dissection	28 (74)	3 (21)

Twenty-one patients (48%) presented with ischemic signs and symptoms (stroke or TIA), 20 patients (45%) presented with local symptoms only, and 3 patients presented with non-specific signs (dizziness in patients with ICA dissection). Seven patients (16%) had multiple dissected vessels with a total of 15 vessels; 7 of the vessels were considered the source of the patients’ symptoms (at the same side of the neck pain, Horner’s syndrome, or the stroke on imaging studies) while the other 8 were considered asymptomatic.

Of the 44 patients, 41 (93%) were treated with aspirin and clopidogrel and 1 with aspirin only (patient preference), 1 declined medical treatment, and 1 was not medically treated due to elevated INR caused by advanced liver cirrhosis. Clinical and imaging follow-up was available for 42 patients (50 vessels). Follow-up averaged 18.1 months (range: 3–108 months). There were no recurrent ischemic events (stroke or TIA), deaths, symptomatic intra-cranial hemorrhage, or major extracranial hemorrhage reported during follow-up. The most common complaint during follow-up was headache in 5 (12%) and neck pain in 2 (5%) of the 43 patients.

Of the 52 vessels, 10 were treated by endovascular techniques (4 stents were placed to reconstitute the vascular lumen, 4 side-wall aneurysms were coiled, and 2 vessels were sacrificed), 5 of which were treated within 6 months of presentation. One vessel was lost to follow-up, leaving 46 vessels on medical treatment alone with at least 6 months of imaging follow-up. Healing, as defined here, occurred in 19/46 vessels (41%), no healing occurred in 27/46 vessels (59%), and one type I dissection progressed to a type II dissection (this was considered no healing). Four type II dissections healed, with an average time to healing of 4.8 months (range: 3–8 months) and all were small side-wall aneurysms. Overall, of the 15 small side-wall aneurysms present, 5 healed on medical therapy alone (33%). Fifteen type I dissections healed completely, with an average time to healing of 4.2 months (range: 3 weeks to 11 months). Data stratified by type I and type II is presented in Table 2.

**Table 2:** **Type I and type II dissections**.

	Type I, *n* = 21 (%)	Type IA, *n* = 15 (%)	Type IB, *n* = 6 (%)	Type II, *n* = 31 (%)	Type IIA, *n* = 24 (%)	Type IIB, *n* = 7 (%)
**CLINICAL PRESENTATION**						
Ischemic symptoms	14 (67)	11 (73)	3 (50)	7 (23)	5 (21)	2 (29)
Local symptoms	5 (24)	2 (13)	3 (50)	15 (48)	15 (63)	0 (0)
Asymptomatic	1 (5)	1 (7)	0 (0)	7 (23)	3 (12)	4 (57)
Non-specific symptoms	1 (5)	1 (7)	0 (0)	2 (6)	1 (4)	1 (14)
**IMAGING EVOLUTION AT 6 MONTHS**						
Healed	15/20 (75)	12/14 (87)^a^	3/5 (60)^b^	4/26 (15)	4/21 (19)^c^	0/5 (0)^d^
No change	4/20 (20)	2/14 (13)^a^	2/5 (40)^b^	22/26 (85)	17/21 (81)^c^	5/5 (100)^d^

^a^One type IA vessel progressed to type II.

^b^One type IB vessel was treated with endovascular techniques within the first 6 months.

^c^Three type IIA vessels were treated with endovascular techniques within the first 6 months.

^d^One type IIB vessel was treated with endovascular techniques within the first 6 months and one was lost to follow-up.

Vertebral artery dissections were predominately type I (11/14, 79%), while ICA dissections were predominately type II (28/38, 74%) (*p* < 0.001). Type I dissections had statistically higher chances to present with ischemic symptoms (14/21, 67%) when compared to type II dissections (7/31, 22.5%) (*p* = 0.001), while Type II dissections had higher chances to present with local symptoms (15/31, 48%) than type I dissections (5/21, 24%), almost reaching a statistical significance (*p* = 0.057). Type I, however, had a statistically higher chance of healing than type II (75 vs. 15%, respectively) (*p* < 0.001).

## Discussion

Spontaneous cervical artery dissection is an important cause of stroke in younger patients accounting for as much as 20% of ischemic stroke in younger patients (11–16). Yet despite its prevalence, its pathogenesis, risk of subsequent ischemic stroke, and best treatment are still debated.

Until recently, dissection was believed to be due to a tear in the intima (inside-out theory) (14). However, new evidence suggests that the pathological process may begin with degenerative changes at the medial-adventitial border associated with neoangiogenesis of capillary vessels branching from the vasa vasorum in the adventitia (outside-in theory) (17, 18). In the BMC-sCAD registry, clear imaging signs of intimal tear were found in only 60% of cases, as seen by contrast extravasation into the vascular wall (Borgess classification type II). Clear intimal flap was found in only 13% of our cases (type IIB). Other authors have reported similarly low percentages of intimal flap on CTA at 25% (19). This observation supports the outside-in theory of arterial dissection, since if the intimal tear is the initial insult, we would expect to encounter intimal tears at a much higher rate. It is conceivable that there could be an initial intimal tear that healed by the time of imaging or went undetected if its extremely small size did not allow for detection of extra-luminal contrast pooling. However, if the original intimal tear is extremely small, there is still not an explanation for how such a large amount of blood enters the arterial wall causing symptomatic dissection and how it is closed so quickly afterward.

In our series, when an intimal tear was present (type II) it rarely resolved (only about 15% of cases). In the few cases in which the intimal tear did resolve, it took approximately 5 months. Therefore, a more logical explanation is that in the cases where no intimal tear is found, there was never one to begin with. Other authors also commented on the rarity of finding evidence of intimal tear/flap (18–20). These arguments point to an initial abnormality at the vascular wall level (outside-in theory) as the most plausible explanation.

Additionally, in our registry most ICA dissections were type II (74%) while the majority of VA dissections were type I (79%), a finding that is difficult to reconcile with the outside-in theory as proposed. If in both cases the dissection starts inside the vascular wall, why is there a higher incidence of intimal tear in the ICA compared to the VA? We believe that this difference may be related to the higher mobility of the ICA in comparison to the VA, which is encased in the cervical vertebrae. This observation argues for a mechanical component (e.g., trauma, sudden twisting) leading to intimal tear, suggesting tears are probably not only due to the extension of the intramural hematoma rupturing the intimal layer as the pure outside-in theory states. A mechanical component could play a role at least in certain circumstances. A modified theory for the genesis of cervical arterial dissection is that the process begins as an outside-in process with formation of an intramural hematoma (type I dissection) which may or may not become symptomatic depending on the degree of vascular stenosis, clot formation, and collateral blood supply. In certain cases, a superimposed minor trauma may occur and intimal tear may develop in this already-dissected vessel; one case of such a conversion from type I to type II occurred in our registry, which further strengthens our belief in this possibility.

The risk of subsequent stroke following sCAD while the patient is on medical therapy (anticoagulant or anti-platelet) has been very low (1–2%) in multiple retrospective series (21–27). This estimation was recently challenged by a prospective study reporting the overall subsequent stroke risk following dissection at 10.4% (12.4% for aspirin, 8.3% for warfarin) (28). Our registry compares favorably with its subsequent stroke rate of 0%, which may be due to the protective effect of the dual anti-platelet therapy and its rapid initiation (as soon as the diagnosis was confirmed). The two types of dissections we describe may have different risks of subsequent stroke. A larger prospective study documenting the real risk of subsequent stroke in these patients and comparing the protective effect of dual anti-platelet vs. ASA or warfarin is certainly long overdue.

Perhaps the most practical impact for this new classification of sCAD is its ability to predict healing following medical therapy alone. Because we performed direct vascular imaging, we were able to define healing as the reconstitution of the vessel lumen to normal anatomy as opposed to defining healing as the persistence or recurrence of antegrade flow as is often reported (12, 21, 29). Reconstitution of antegrade flow was observed in 45/46 (98%) dissections on follow-up. However, when looking at true healing, as opposed to mere antegrade flow, a substantially higher percentage of type I dissections healed compared to type II (75 vs. 15%, *p* < 0.001). Type IA dissections had a better rate of healing (80%) compared to type IB (60%). Type IIA dissections healed only 19% of the time, while no type IIB healed (0%). When comparing the size of the type IIA side-walled aneurysms, 5/15 vessels with a small aneurysm healed (33%), while no large aneurysms healed (0/10). This data suggests that type I dissections undergo substantial remodeling/healing while type II dissections do not. This different healing rate may in part be dependent on the presence of an intimal tear, the extent of the tear, and the preservation of antegrade flow. In agreement with other studies, we found that if healing does occur, it will occur within the first 6 months (29).

This paper has limitations. The absence of an independent observer to assess the recurrent stroke rate is one limitation, as is the study’s sample size. The more significant limitation is that the classification is based on the presence or absence of intimal tear as depicted by current available imaging technologies, which are low resolution for examining the intima. However, this limitation is unavoidable given that there are no other ways to make this distinction short of autopsy, which would obviously limit the classification’s use in treatment and prognosis. Therefore, despite these limitations this classification could be useful.

After analyzing this registry, we have adjusted our clinical practice. Dual anti-platelet therapy is the first-line of treatment with clinical and imaging follow-up at 6 months. The risk of subsequent stroke and further remodeling of the vascular wall are both extremely low. We reserve endovascular techniques for specific cases, such as to stent a dissected vessel when the CBF has decreased beyond the compensatory capability of the collateral blood supply. The endovascular approach could also be useful for treating large side-walled aneurysms with persistent local symptoms. The exact place for endovascular treatment is still not clear; however, using this classification, a more rational recommendation could be put forward beyond the “indicated or not indicated” dichotomy.

## Conclusion

We believe that the Borgess classification of sCAD helps to plan treatment and predict healing following medical treatment. The registry supports a modified outside-in theory of pathogenesis of sCAD. Dissections without intimal tear (type I) had a higher rate of healing than dissections with intimal tear (type II). Dual anti-platelet therapy appears to be beneficial in reducing the risk of subsequent stroke. A large, prospective, randomized trial is needed to define the best medical treatment for sCAD.

## Conflict of Interest Statement

The authors declare that the research was conducted in the absence of any commercial or financial relationships that could be construed as a potential conflict of interest.
